# Bees do not use nearest-neighbour rules for optimization of multi-location routes

**DOI:** 10.1098/rsbl.2011.0661

**Published:** 2011-08-17

**Authors:** Mathieu Lihoreau, Lars Chittka, Steven C. Le Comber, Nigel E. Raine

**Affiliations:** 1 Biological and Experimental Psychology Group, School of Biological and Chemical Sciences, Queen Mary, University of London, London E1 4NS, UK; 2 School of Biological & Chemical Sciences, Queen Mary, University of London, London E1 4NS, UK

**Keywords:** *Bombus terrestris*, foraging routes, pollination ecology, spatial cognition, trapline foraging, travel optimization

## Abstract

Animals collecting patchily distributed resources are faced with complex multi-location routing problems. Rather than comparing all possible routes, they often find reasonably short solutions by simply moving to the nearest unvisited resources when foraging. Here, we report the travel optimization performance of bumble-bees (*Bombus terrestris*) foraging in a flight cage containing six artificial flowers arranged such that movements between nearest-neighbour locations would lead to a long suboptimal route. After extensive training (80 foraging bouts and at least 640 flower visits), bees reduced their flight distances and prioritized shortest possible routes, while almost never following nearest-neighbour solutions. We discuss possible strategies used during the establishment of stable multi-location routes (or traplines), and how these could allow bees and other animals to solve complex routing problems through experience, without necessarily requiring a sophisticated cognitive representation of space.

## 1. Introduction

Complex problem solving is often seen as an indicator of advanced intelligence requiring causal reasoning and a brain with large processing power [1]. However, animals sometimes perform remarkably well using simple decision rules [2]. This is perhaps best demonstrated by considering the case of routing decisions animals face when exploiting patchily distributed resources. These optimization problems are analogous to the well-known travelling salesman problem, in which the task is to find the shortest route that passes through a set of locations (visiting each only once) before returning to the start [3]. The only way to find the shortest route is to measure and compare the lengths of all possible routes, which becomes increasingly difficult for large sets of locations since the number of possible routes increases factorially with the number of locations to visit. However, approximations of the optimal solution can be obtained within a reasonable time using heuristic procedures and computer simulations [4]. Therefore, rather than comparing all routes, animals are assumed to rely on simple heuristics coupled with spatial memory [5]. For instance, humans navigating between multiple locations tend to move to the nearest available unvisited location (or clusters of locations) until all have been visited [6]. This observation is congruent with findings from non-human primates [7,8], rats [9] and bees [10,11], suggesting that foraging animals find functional routes by linking nearest-neighbour resources. Such a movement rule may facilitate the establishment of stable and repeatable multi-location routes or ‘traplines’ [12], a taxonomically widespread behaviour known to increase foraging efficiency [13]. While animals are able to modify their traplines in response to changes in both the distribution [14] and quality [15] of resources, the underlying optimization process remains unknown.

To test whether bees rely on nearest-neighbour movements, we investigated the travel optimization performance of traplining bumble-bees faced with a multi-location routing problem in a flight cage. Bees were observed foraging on six artificial flowers arranged such that movements between nearest-neighbour flowers would lead to a long suboptimal route.

## 2. Material and methods

### (a) Subjects

Workers from a commercially obtained *Bombus terrestris* colony (Syngenta Bioline Bees, Weert, The Netherlands) were marked with individually numbered tags within 1 day of emergence. The colony was provided with ad libitum pollen. Workers collected sucrose solution (40% w/w) from artificial flowers.

### (b) Artificial flowers

Experiments were performed in an indoor flight room (870 × 730 × 200 cm) with controlled illumination (for details see Lihoreau *et al*. [14]). The nest-box and six artificial flowers were placed in different locations (electronic supplementary material, figure S1*a*). Each flower consisted of a blue landing platform sitting on a sucrose reservoir, from which a feeding cup was accessible to the bees (electronic supplementary material, figure S1*b*). Flowers were refilled using a remote control box. Spatial arrangement of the flowers maximized the discrepancy between the distance a bee would fly when following a ‘nearest-neighbour’ strategy (starting and ending at the nest, the bee flies to the nearest unvisited flower until all flowers are visited) or an ‘optimal’ strategy (starting and ending at the nest, the bee visits all the flowers once using the shortest possible route: electronic supplementary material, figure S1*a*). The array provided one unique nearest-neighbour route and two optimal routes (clockwise or anti-clockwise). Posters on the walls acted as unique landmarks to help bees navigate (electronic supplementary material, figures S1*a* and S2).

### (c) Procedure

Prior to tests, bees were allowed to forage ad libitum on the six flowers (feeding cup capacity: 5 µl sucrose solution per flower) placed in a linear patch 1 m in front of the nest entrance and refilled after each visit. After 2 h, each forager was observed for three additional foraging bouts and the volume of sucrose ingested during each of these bouts was used to estimate its crop capacity (range: 120–180 µl). Using this information, we set the rewards provided by each flower to one-sixth of each individual's crop capacity during experiments, so that a worker feeding from all six flowers would fill its crop within a foraging bout. Test bees were selected to minimize variations in age and body size (*n* = 8; age: mean ± s.e. = 9.25 ± 2.18 days; thorax width: mean ± s.e. = 5.23 ± 0.10 mm). They were observed individually for 80 foraging bouts on the same day in the six-flower arrangement (electronic supplementary material, figure S1*a*). Flowers were refilled after each foraging bout. For each bout, we recorded the order in which the bee landed on each flower and the time of each visit. The distance flown was calculated as the minimum distance flown in a straight line between flowers. Between testing bees, flowers were washed (70% ethanol) to remove scent marks.

### (d) Data analysis

We analysed the travel optimization performance of bees (flight distances, flight durations and number of flower visits) using complete flower visitation sequences, including all revisits to the same flower. To analyse the geometry of routes, we focused on the first visit to each flower only [14,15]. Excluding revisits does not influence the overall geometry, as the clear majority (69.75% of revisits, *n* = 3696) were immediate returns to the flower just visited (not different locations) and revisit frequency drops sharply with experience (see §3). Furthermore, a bee moving between nearest-neighbour flowers and making ad libitum revisits would never follow the shortest possible (convex-hull) route. Assuming that there are 720 (6!) possible routes to visit all flowers once, we explored the frequency of route usage using multinomial tests with a random probability of 1/720. We assessed the directionality of bees (tendency to move in a constant direction between flowers) by comparing the number of clockwise and anti-clockwise sequences with binomial tests. We excluded from analyses bouts in which bees did not visit all six flowers. Most of these bouts occurred in inexperienced bees (greater than 50% in the first 10 bouts, electronic supplementary material, table S1) and were equally distributed among bees (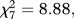 *p* = 0.26, mean ± s.e. = 10.31 ± 1.54). Means are given with standard errors.

## 3. Results

### (a) Travel optimization performance

**Figure 1.**
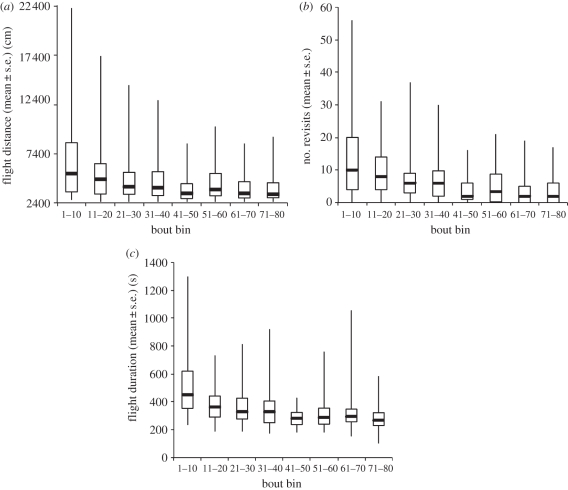
Travel optimization performance. Box plots indicate (*a*) average flight distances, (*b*) number of revisits to flowers, and (*c*) flight durations per bin of 10 foraging bouts (*n* = 8 bees). In each box, the thick horizontal bar is the median, while the lower and upper edges represent the 25% and 75% quartiles, respectively. Whiskers indicate the maximum and minimum values that are not outliers.

Bees significantly reduced their flight distances as they gained experience with the flower array (generalized linear mixed model (GLMM), *t*_576_ = 8.81, *p* < 0.01), from a mean distance of 6541 (±1354) cm in the first 10 bouts (165.68 ± 55.02% longer than optimal route length) to 3840 (±512) cm in the last 10 bouts (55.96 ± 20.78% longer than optimal route length; figure 1*a*). This twofold reduction in flight distance was accompanied by a decrease in the number of revisits to empty flowers (GLMM, *t*_576_ = 10.29, *p* < 0.01; figure 1*b*), and the flight duration (GLMM, *t*_576_ = 11.20, *p* < 0.01; figure 1*c*).

### (b) Spatial geometry of routes

**Figure 2.**
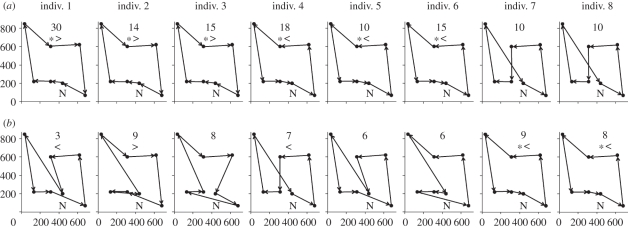
Spatial geometry of routes. Scale diagrams represent (*a*) the primary and (*b*) the secondary route for each bee (*n* = 8). Filled circles are flowers, N is the nest-box and arrows indicate the direction in which the bee moved. The number above each panel is the frequency with which the route was used during 80 trials. Asterisks (*) indicate an optimal route, greater than symbols (>) indicate clockwise routes and less than symbols (<) indicate anti-clockwise routes. Axis labels are given in centimetres.

Each bee used an average of 10.75 (±0.99) different routes, significantly more than expected by chance (electronic supplementary material, table S1). Six of the bees selected an optimal route as their primary (most frequently used) route and the other two selected an optimal route as their secondary route (figure 2). On average, they started using the shortest possible route after completing 26.63 (±4.88) foraging bouts and used it in 21.41 per cent (±2.96) of all bouts (figure 2). In contrast, they flew the nearest-neighbour route in only 0.31 per cent (±0.20) of their bouts and none of them used it more often than expected by chance. Analysis of choice sequences, irrespective of which flower was visited first, confirmed that bees showed no tendency to move between nearest-neighbour flowers (only 4.06 ± 1.13% of all bouts; table 1). Bees also did not optimize the geometry of routes when the first flower they visited was neither flower 1 nor 6 (3.29 ± 1.46% of all bouts; table 1). However, each bee exhibited a biased directionality of movements (binomial test, *p* < 0.05 for 7 bees; figure 2), with a consistent tendency to visit the flowers either in a clockwise or anti-clockwise sequence. The fact that bees showed no tendency to visit flowers close to the flight cage perimeter more often than flowers nearer the centre (electronic supplementary material, figure S3), suggests it is unlikely that bees engaged in wall-following behaviour.

**Table 1.** Average percentage of foraging bouts (mean ± s.e., *n* = 8 bees) in which bees followed the optimal travel distance or linked flower visits by making nearest-neighbour movements in relation to the first visited flower. Numbers in parentheses indicate the travel distance for each sequence. Bees did not follow an optimal route if their first visit was not to either flower 1 or 6. Wilcoxon tests (*p*-values) were used to compare the percentage of bouts starting at each flower in which bees followed an optimal route or the nearest-neighbour route.

first visited flower	optimizing overall travel distance		linking nearest-neighbour flowers		
	sequence	% of bouts	sequence	% of bouts	*p*-value
1	123 456 (2462 cm)	11.41 ± 1.41	124 563 (3380 cm)	0.31 ± 0.06	0.02
2	213 456 (2692 cm)	1.41 ± 0.25	214 563 (3518 cm)	0	0.17
3	345 621 (2889 cm)	0.16 ± 0.05	345 126 (2984 cm)	1.56 ± 0.34	0.27
4	453 216 (2855 cm)	0.16 ± 0.05	451 263 (3771 cm)	0.47 ± 0.07	0.42
5	543 216 (2633 cm)	1.56 ± 0.29	543 216 (2633 cm)	1.56 ± 0.29	1
6	654 321 (2462 cm)	10 ± 0.90	612 453 (2985 cm)	0.16 ± 0.05	0.01

## 4. Discussion

Most bees minimized their overall travel distances by selecting the shortest possible path as a trapline. Although previous studies suggested that bees use a nearest-neighbour rule to develop multi-location routes, flower arrangements were not specifically designed to test this hypothesis [10,11]. Our results clearly demonstrate that they do not rely on such a rule when it produces a profoundly suboptimal outcome.

So how do bees optimize their routes between flowers? The fact that bees gradually reduced their travel distances with experience indicates that the optimization process relies on learning and spatial memory of flower locations. Flower visitation sequences also suggest that the acquisition of short traplines could be facilitated by a consistent directionality of movements. Stable flight direction when leaving a flower may initially generate straight route segments and a tendency to choose the next flower visited by its proximity to the current angular bearing of travel. In this experiment, such a strategy would favour the establishment of circular routes around the edge of the array in combination with path integration and spatial memory of flower locations [16,17]. Gradual optimization of route length may then occur with experience (by trial-and-error) by comparing the length of the current route to those previously explored, possibly using differences in the rates of optic flow [18]. Such an optimization process is very similar to those implemented in ‘convex-hull’ heuristics (which calculate the minimum polygon containing the entire set of points in an array [4]). These heuristics from computer sciences have been proposed to explain human optimization performance when connecting dots on a computer screen in visual versions of the travelling salesman problem [19]. Whether animals use this movement rule when navigating between distant locations remains to be tested. Convex-hull heuristics could provide a parsimonious explanation to complex routing problem solving by traplining animals, without necessarily requiring a sophisticated cognitive representation of space.
